# Co-Existing Nanoplastics Further Exacerbates the Effects of Triclosan on the Physiological Functions of Human Serum Albumin

**DOI:** 10.3390/life15010112

**Published:** 2025-01-16

**Authors:** Yan Bao, Yaoyao Wang, Hongbin Liu, Jing Lan, Zhicai Li, Wansong Zong, Zongshan Zhao

**Affiliations:** 1 Shandong Key Laboratory of Water Pollution Control and Resource Reuse, School of Environmental Science and Engineering, Shandong University, Qingdao 266237, China; 2 Qingdao Institute of Bioenergy and Bioprocess Technology, Chinese Academy of Sciences, Shandong Energy Institute, Qingdao New Energy Shandong Laboratory, Qingdao 266101, China; 3 School of Environment and Geography, Qingdao University, Qingdao 266071, China; 4 Anqiu Branch of Weifang Municipal Bureau of Ecology and Environment, Weifang 262199, China; 5 College of Geography and Environment, Shandong Normal University, Jinan 250014, China

**Keywords:** human serum albumin, interaction mechanism, nanoplastics, triclosan

## Abstract

The potential health risks posed by the coexistence of nanoplastics (NPs) and triclosan (TCS) have garnered significant attention. However, the effects and underlying mechanisms of NPs and TCS on key functional proteins at the molecular level remain poorly understood. This study reports the effect of polystyrene nanoplastics (PSNPs) on the binding of TCS to human serum albumin (HSA) using multispectral methods and molecular simulation systems. The experimental results show that TCS significantly inhibits HSA esterase activity, with exacerbating inhibition in the presence of PSNPs, which is attributed to the alteration of HSA conformation and microenvironment of the amino acid residues induced by PSNPs. Molecular docking and site marker competitive studies indicate that TCS predominantly binds to site I of subdomain Sudlow II and the presence of PSNPs does not affect the binding sites. Spectra analyses indicate that the quenching mechanism between TCS and HSA belongs to the static quenching type and the presence of PSNPs does not change the fluorescence quenching type. The HSA fluorescence quenching and the conformational alterations induced by TCS are further enhanced in the presence of PSNPs, indicating that PSNPs enhance the binding of TCS to HSA by making TCS more accessible to the binding sites. This study provides valuable information about the toxicity of PSNPs and TCS in case of co-exposure.

## 1. Introduction

In contemporary society, plastics are extensively utilized in various sectors, including commercial, industrial, and municipal domains. In 2022, the global production of plastics exceeded 430 million tons. Although plastics simplify our lives, their leakage has presented detrimental effects on the environment and human health. Over time, plastic products exposed to environmental factors, such as high temperature, oxidation, wear, and ultraviolet light, gradually break down into smaller pieces, eventually forming microplastics (MPs) and nanoplastics (NPs) [1,2]. Common plastics, mainly polyethylene (PE), polypropylene (PP), polyvinyl chloride (PVC), and polystyrene (PS), are degraded to MPs and NPs [3]. MPs and NPs, defined as particles smaller than 5 mm and 0.1 μm, respectively [4], are ubiquitous and frequently detected in various environments, including soils, oceans, lakes, rivers, sediments, and living organisms [5]. Consequently, MPs and NPs have garnered significant attention and become a prominent research field in recent years [6].

These small plastics can enter organisms through various routes, including water and food [7], and accumulate along the food web [8,9]. Due to their small particle sizes, large specific surface area, and high hydrophobicity [10], MPs and NPs pose greater hazards to the environment and human body compared to larger plastic debris. Numerous studies have shown intensified or weakened toxic effects of co-existence with MPs and certain toxic pollutants, such as persistent organic pollutants and heavy metals [11]. For instance, exposure to polystyrene microplastics and sulfamethoxazole antibiotics resulted in a significant accumulation in detoxification organs, such as the liver, leading to increased oxidative and inflammatory damage compared to exposure to sulfamethoxazole alone [12]. Similarly, NPs have a high adsorption capacity for organic pollutants [13,14]. Further investigations are needed to study the complex effects of NPs when co-existing with other contaminants.

Triclosan (2,4,4′ -trichloro-2′-hydroxydiphenyl ether, TCS) is a wide-spectrum antibacterial agent known for its excellent antibacterial qualities and ease of synthesis [15]. It is widely used in various medications and personal care products, including soaps and hand sanitizers [16]. The widespread use of personal care products has resulted in significant releases of triclosan into the environment. Triclosan is widely present in nature and the human body (e.g., breast milk, fat, and blood) [17]. Triclosan can accumulate in the human body, resulting in liver and kidney toxicity, thyroid dysfunction, and damage to the nervous and reproductive systems [18,19]. Despite policies prohibiting its use in personal care products, TCS remains in widespread use in other fields [20].

The co-existence of NPs and TCS has been a concern in recent years. A recent study found that the combined exposure to NPs and TCS significantly affected male zebrafish, leading to decreased sperm quality and disrupted endocrine function [21]. More importantly, the combined exposure of NPs and TCS produces synergistic toxic effects on humans, resulting in severe cell damage and dysfunction [22]. Most previous studies focused on the combined effects of NPs and TCS at the cellular level. However, the combined effects on proteins are still not clearly illustrated.

Human serum albumin (HSA), the most abundant protein in human plasma [23], has a strong ligand-binding capacity and can transport a variety of endogenous and exogenous substances to specific targets, such as fatty acids, hormones, and drugs [24]. HSA is a non-carbonylated single-chain protein molecule composed of 585 amino acids, consisting of three structurally similar domains: domain I (1–95), domain II (196–383), and domain III (384–585), each comprising two subdomains (A and B) [25,26]. The primary drug-binding sites are located in the Sudlow I site in the IIA domain and the Sudlow II site in the III A domain. Thus, HSA is an ideal protein model for studying the interactions between small molecules and proteins [27]. Since NPs and TCS are consistently detected in human blood, their coexistence may cause immeasurable harm to the human body. Hence, studying the functional and conformational changes in HSA proteins induced by TCS and NPs is essential for comprehending their adverse impacts on human health.

PS has gained widespread application in packaging materials, household appliances, disposable tableware, and other daily life domains, thereby its degradation into MPs and NPs greatly increases the risk of human exposure [28]. In this study, polystyrene nanoplastics (PSNPs) were chosen to study their effects on TCS and HSA binding. The effects of TCS on the relative esterase activity and conformation of HSA in the presence of polystyrene nanoplastics (PSNPs) were studied by using fluorescence quenching, synchronous fluorescence spectroscopy, UV-vis spectroscopy, three-dimensional (3D) fluorescence spectroscopy, and circular dichroism (CD) spectroscopy [29,30]. Computer simulations provided the reasonable structure of the HSA-TCS complex and identified important amino acids in the binding process [31]. This study is the first to elucidate the effects and underlying mechanisms of PSNPs on TCS-induced toxicity of HSA, providing valuable information about the complicated behavior of NPs coexisting with organic pollutants.

## 2. Materials and Methods

### 2.1. Materials

HSA (purity > 98%) was obtained from Biotopped Technology Co., Ltd. (Beijing, China). TCS (purity > 99%) was purchased from Yuanye Biotechnology (Shanghai, China). PSNPs with a diameter of 100 nm were purchased from Wuxi Ruger Biotechnology Co., Wuxi, China. Ibuprofen (purity > 98%) and phenylbutazone (purity > 98%) were purchased from Eon Chemical Technology (Shanghai, China). HSA stock solution (1 × 10^−5^ mol/L) was configured by dissolving 33 mg of HSA powder in 100 mL of ultrapure water. TCS stock solution (1 × 10^−4^ mol/L) was made by mixing ultrapure water and HPLC-grade methanol. PSNPs were dissolved in ultrapure water with sonication to prepare the PSNPs dispersion. A phosphate buffer solution (pH = 7.4) was utilized in all experiments to keep the pH. Store all solutions at 0–4 °C protected from light until use. Currently, the exposure concentrations of TCS and NPs in the aquatic environments are 0.0034–13 μM and 1.53–13.8 μg/mL, respectively [32,33]. Therefore, the concentration range of TCS was set to 0–12 μM, and the concentration of PSNPs was set to 3 μg/mL, which better represents the effects of TCS and PSNPs on HSA in the aquatic environment.

### 2.2. Esterase-like Activity Experiments

The effects of TCS and PSNPs on the HSA esterase-like activity were assessed using p-nitrophenyl acetate (p-NPA) as the substrate [34]. TCS (0–8 μM) and PSNPs (3 μg/mL) were added to the HSA solution (1 μM) and incubated for 1 h. The substrate solution (5 × 10^−3^ mol/L) was then added to the mixture and absorbance at 405 nm was immediately monitored using a Shimadzu UV-1780 spectrophotometer (Tokyo, Japan) for 300 s. The percentage of relative esterase activity was calculated as follows [35,36]:

(1) $$\mathrm{Inhibition}\;(\%)=A_{i}/A_{0}$$

(2) $$∆A=A_{300s}-A_{0s}$$

where $∆A_{i}$ represents the absorbance change from 0 to 300 s after adding the mixed HSA solution containing PANPs and TCS, and $∆A_{0}$ is the absorbance change from 0 to 300 s after adding the HSA solution without PANPs and TCS.

### 2.3. Fluorescence Spectral Measurements

Fluorescence measurements were performed using an F-2710 fluorescence spectrophotometer (Hitachi, Tokyo, Japan) equipped with a 150 W xenon lamp and a 1.0 cm quartz cell. The absorbance at emission and excitation wavelengths were maintained at 0.1 to avoid the filter effect. Fluorescence quenching spectra for the interaction between HSA and TCS in the absence or presence of PSNPs were recorded. The solutions for each group were thoroughly mixed and allowed to react for 1 h at 293 K. The excitation wavelength was set at 280 nm, and the emission wavelength range was 310–400 nm, with a slit width of 5 nm. The fluorescence intensity of PSNPs was also measured with this wavelength range to correct the experimental results. The concentration of HSA was constant at 1 μM, while the concentration of TCS varied from 0 μM to 12 μM, with a fixed PSNPs concentration of 3 μg/mL.

### 2.4. Molecular Docking Studies

MOE 2008.10 was utilized to simulate the binding mechanism of small compounds to proteins (Montreal Chemical Computing Ltd., Montreal, QC, Canada). Molecular conformations of HSA were taken from Protein Data Bank (https://www.rcsb.org/ (accessed on 20 March 2024)) (ID: 1BJ5, resolution: 2.50 Å). The molecule structures of TCS were taken from PubChem (https://pubchem.ncbi.nlm.nih.gov/ (accessed on 20 March 2024)). Before docking, HSA was protonated, bound, and minimized, and unbound water was removed. The optimal interactions between TCS and HSA were identified by screening using root mean square deviation (RMSD) values [37]. The optimal binding result was determined by the lowest S-score. The software Chimera 1.16 (https://www.cgl.ucsf.edu/chimera/ (accessed on 28 March 2024)) was used to visually evaluate the docked conformations.

### 2.5. Site Marker Competitive Experiments

The site competition assay was performed using site markers phenylbutazone (site I) and ibuprofen (site II) [38,39]. The fluorescence intensity values at the emission wavelength of 340 nm were measured at room temperature using a HITACHI F-2700 spectrophotometer (Hitachi, Japan). The concentrations of HSA and TCS were kept constant at 1:10, and the site markers concentration (0–12.5 μM) was gradually increased to titrate the complexes of HSA-TCS and HSA-TCS-PSNPs.

### 2.6. Synchronous Fluorescence Studies

The synchronized fluorescence scan at Δλ = 60 nm was performed with λ_ex_ of 280 nm and λ_em_ ranging from 220 to 320 nm. Another scan at Δλ = 15 nm was performed with λ_ex_ = 280 nm and λ_em_ ranging from 265 to 320 nm. In this wavelength range, the fluorescence signal displayed by TCS is very weak and can be neglected [40]. The final spectra of the interacting systems were corrected by subtracting the emission spectra of PSNPs from those of HSA-TCS-PSNPs [39].

### 2.7. UV–Vis Spectrophotometry

Absorption spectra of TCS (0–12 μM) and HSA (1 μM) with or without PSNPs (3 μg/mL) were recorded in the wavelength range from 200 to 400 nm using a Shimadzu UV-1780 spectrometer (Shimadzu, Japan) at room temperature. The background absorptions of TCS and PSNPs were excluded from the measurements [25].

### 2.8. 3D Spectral Studies

The 3D fluorescence spectra of HSA-TCS and HSA-TCS-PSNPs systems were recorded using a HITACHI F-2700 spectrophotometer (Hitachi, Japan) to explore the conformation changes of HSA. The excitation wavelengths were 200–400 nm, and the emission wavelengths were 200–500 nm [41]. The ratio of HSA to TCS concentrations was fixed at 1:2, and the concentration of PSNPs was fixed at 3 μg/mL.

### 2.9. CD Studies

CD spectra of HSA (1 μM) with or without TCS (2 μM) and PSNPs (3 μg/mL) were recorded using the Jasco J-1500 spectropolarimeter (Tokyo, Japan) CD chromatograph in the wavelength range from 200 to 260 nm at a scanning rate of 200 nm/min. The system was continuously flushed with nitrogen. Two parallel scans were performed for each spectrum [42]. BeStSel, an online program, was used to quantitatively analyze the CD spectra.

## 3. Results and Discussion

### 3.1. Effect of TCS and PSNPs on HSA Esterase Activity

**Figure 1:**
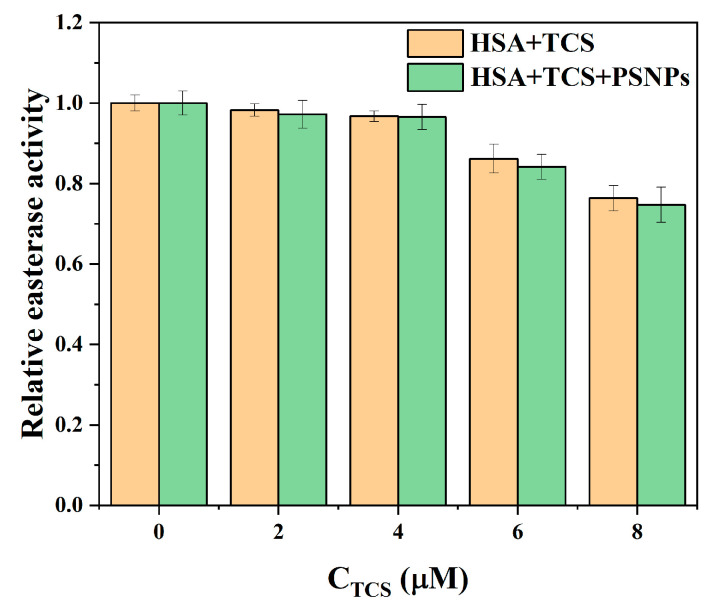
Effect of TCS on relative esterase activity of HSA in the absence and presence of PSNPs. [HSA] = 1 μM, [PSNPs] = 3 μg/mL, and T = 293 K.

The hydrolytic function of HSA, similar to esterase, is crucial for its physiological role [43]. The effects of TCS and PSNPs on the structure of HSA were explored by the esterase-like activity of HSA. In the HSA-TCS system, the HSA esterase activity decreased as the concentration of TCS increased (Figure 1). When the concentration of TCS reached 8 μM, the esterase activity was reduced by 24% (Figure 1). This finding indicates that TCS inhibits HSA esterase activity by binding to the enzyme-substrate complex, thereby preventing the conversion of the substrate (p-NPA) to the product (p-nitrophenol) [44]. In the presence of PSNPs, the decrease in HSA esterase activity was even more pronounced, with a 30% reduction observed at 8 μM TCS (Figure 1). This result demonstrates that the combined effects of PSNPs and TCS further inhibit the esterase activity of HSA. However, there was little change in esterase-like activity with the addition of PSNPs alone ([TCS] = 0 μM; Figure 1) [45,46], indicating PSNPs have minor effects on the HSA-activated site. It should be noted that PSNPs can interact with HSA and lead to changes in protein conformation [45]. PSNPs exhibit weak adsorption effects on TCS [47]. Therefore, the combined effect of PSNPs and TCS further decreased the esterase activity of HSA, which may prevent p-NPA from binding to the activated site of HSA by changing the HSA protein conformation and microenvironment [31].

### 3.2. Fluorescence Spectra Analysis

#### 3.2.1. Fluorescence Quenching Study

**Figure 2:**
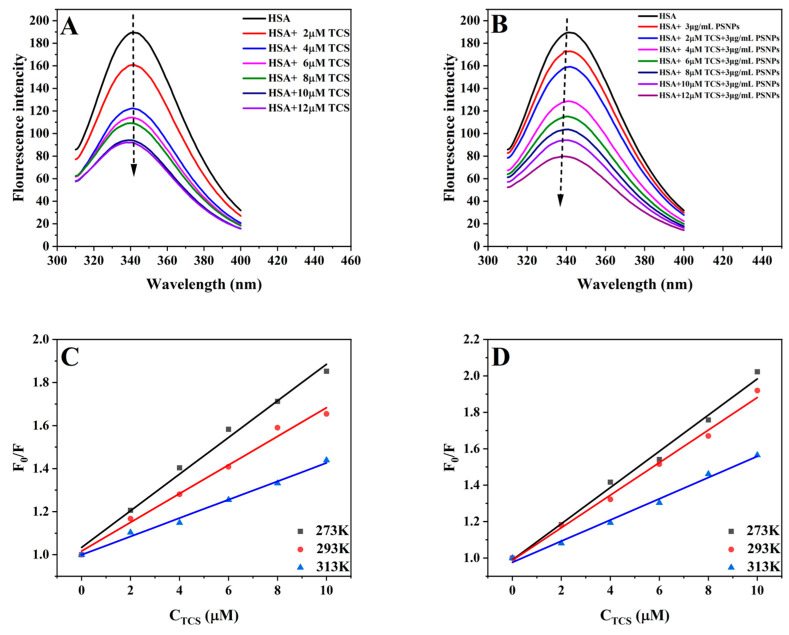
Emission spectra (**A**,**B**) and Stern–Volmer plots (**C**,**D**) of HSA-TCS interactions in the absence (**A**,**C**) and presence (**B**,**D**) of PSNPs. [HSA] = 1 μM and [PSNPs] = 3 μg/mL.

Since protein fluorophores are significantly impacted by small molecule-protein binding, fluorescence spectroscopy provides a reliable method for examining small molecule-protein interactions [48]. Tryptophan (Trp), tyrosine (Tyr), and phenylalanine (Phe) are the three major fluorophores of HSA [49,50]. Because Phe residues have a low quantum yield [51,52], Tyr and Trp residues are the major contributors to the HSA intrinsic fluorescence [53]. Figure 2 presents the fluorescence quenching spectra of HSA and TCS interactions when PSNPs are present and absent. In the absence of PSNPs, the HSA fluorescence intensity decreased gradually with increasing TCS concentrations, while its maximum emission wavelength remained fixed at 333 nm without change (Figure 2A). This indicates that TCS quenches the HSA fluorescence without altering the microenvironment of the fluorophore. PSNPs also had a significant quenching effect on HSA (Figure 2B), consistent with previous research [34,54]. When the concentration of PSNPs was fixed at 3 μg/mL, the HSA fluorescence intensity decreased gradually as TCS concentrations increased (from 0 to 12 μM), accompanied by a 3 nm blueshift (Figure 2B). Compared to the HSA-TCS system, there was a 17% increase in quenching degree in the HSA-TCS-PSNPs system. This indicates that the interactions between HSA and PSNPs may change the hydrophobic microenvironment of the fluorophore [45,52], enhancing the binding of TCS to HSA by making TCS more accessible to the binding sites.

#### 3.2.2. Fluorescence Quenching Mechanism

Fluorescence quenching reflects the reduction of fluorescence quantum yield of fluorophores, caused by various molecular interactions, such as excited state reaction, energy transfer, ground state complex formation, and collision quenching [55]. The quenching mechanism is divided into two types: dynamic quenching and static quenching [56,57]. The former represents the energy transfer caused by the collision between the quencher and the fluorescent substance. The latter refers to the non-fluorescent ground state complex formed by combining the quencher and the fluorescent substance [58]. The main difference between the two quenching mechanisms is their temperature. The quenching rate constant increases with temperature in dynamic quenching, while it decreases with temperature in static quenching [59,60]. Therefore, the fluorescence quenching mechanism of the interaction between TCS and HSA with or without PSNPs could be evaluated using the Stern–Volmer equation at 273 K, 293 K and 313 K temperatures [61]:

(3) $$\frac{F_{0}}{F}=1+K_{q}\tau_{0}\left[Q\right]=1+K_{sv}\left[Q\right]$$

where ***F*_0_** and ***F*** represent the fluorescence intensities of HSA without and with of TCS, respectively. $K_{sv}$ represents the Stern–Volmer quenching constant. $K_{q}$ represents the fluorescence quenching rate constant. $\tau_{0}$ represents the average lifetime of HSA without the presence of the quencher (10^−8^ s), and $\left[Q\right]$ represents the TCS concentration.

Figure 2 presents the Stern–Volmer diagram of the interaction between HSA and TCS at different temperature with and without PSNPs, with the resulting $K_{sv}$ and $K_{q}$ values recorded in Table 1. The $K_{sv}$ value gradually decreased with increasing temperature (Figure 2C) and the $K_{q}$ value was significantly higher than the scattering collision quenching constant (2.0 × 10^12^ M/s) in the absence of PSNPs (Table 1). Therefore, we can conclude that the fluorescence quenching of HSA induced by TCS is likely due to the complex formation rather than dynamic collision [31], indicating a static quenching mechanism. In the presence of PSNPs, the $K_{sv}$ and $K_{q}$ values also decreased as the temperature increased (Figure 2D) but were higher than those at the same temperatures in the absence of PSNPs (Table 1). This suggests that PSNPs do not change the fluorescence quenching type, but they enhance the binding of TCS to HSA and fluorescence quenching efficiency.

### 3.3. The Conformational and Amino Acid Microenvironment Changes of HSA

#### 3.3.1. Molecular Docking Studies

**Figure 3:**
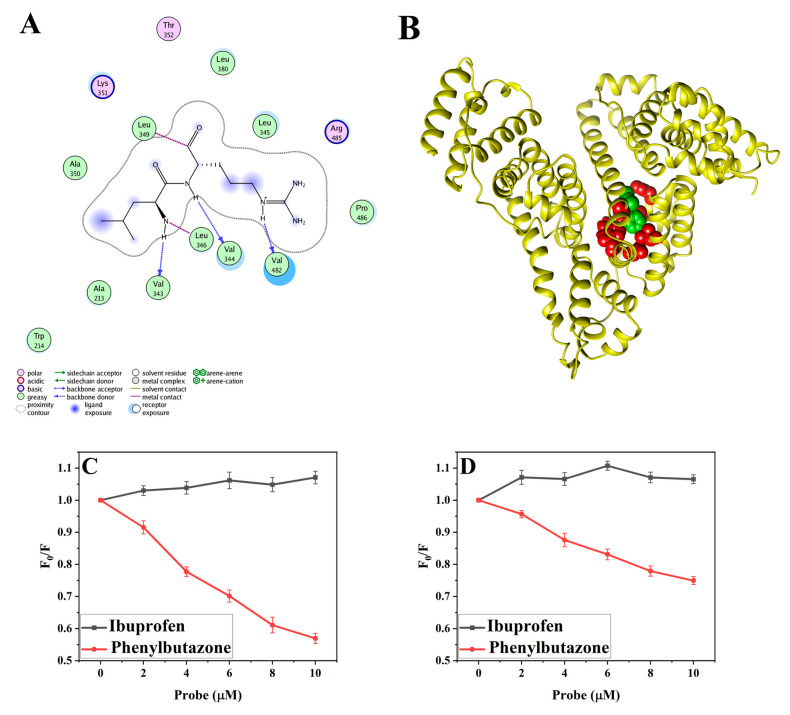
(**A**) 2D diagram of interaction types between TCS and amino acid. (**B**) The binding model of TCS on HSA. Interaction diagram of TCS-HSA under different probe conditions in the absence (**C**) and presence (**D**) of PSNPs: [HSA] = 1 μM, [TCS] = 10 μM, [PSNPs] = 3 μg/mL, and T = 293 K.

Molecular docking studies were conducted to reveal the binding mechanism between TCS and HSA [31,62]. The residue distribution at the intermolecular interaction site and the optimal energy conformation of the TCS-HSA intermolecular interaction are shown in Figure 3A,B [63]. The results show that TCS molecules entered the interior of the HSA active site pocket and were surrounded by amino acid residues. The results demonstrated that TCS interacts with hydrophobic amino acids Ala 350, Trp 214, Ala 213, Val 343, Leu 349, Leu 346, Val 344, Val 482, Leu 380, Leu 345, Pro 486, and hydrophilic amino acids Thr 352, Lys 351, and Arg 485 to form the active site pocket. This suggests that the binding region of TCS and HSA may be located in site I of subdomain Sudlow II [64]. In conjunction with the enzyme activity assay, it was further confirmed that TCS penetrates the active site of HSA, disrupts the microenvironment of key amino acids crucial for esterase function, and results in alterations to the local hydrogen bond network and hydrophobic environment [65]. Since Trp214 is the primary contribution to HSA fluorescence [66], the hydrophobic interaction between Trp214 and TCS is the main reason for the quenching of HSA fluorescence.

#### 3.3.2. Site Marker Competitive Experiment

The competitive binding assay is considered the most direct method to determine the exact binding sites of small molecules on HSA [67,68]. The Sudlow I site in the II A domain and the Sudlow II site in the III A domain are the two hydrophobic active sites of HSA that can bind small molecules [69,70]. Site I contains two aromatic amino acid residues (Tyr and Trp), while site II contains only Tyr residues [71]. This study used ibuprofen and phenylbutazone as representative drugs to identify ligand-binding active sites. Phenylbutazone was used to determine the binding region of site I in subdomain II A, while ibuprofen was used to identify the critical area of site II in subdomain III A [38,72]. In the HSA-TCS system, the absorbance with the addition of ibuprofen remained unchanged as the probe concentration increased, while the absorbance with the addition of phenylbutazone decreased by 45% (Figure 3C). This indicates a competitive relationship between TCS and phenylbutazone at site I, suggesting that the binding site of TCS and HSA is at site I. These findings are consistent with our molecular simulations, which show that the binding site of TCS on HSA is at site I. In the HSA-TCS-PSNPs system, the absorbance after adding ibuprofen remained nearly unchanged, while the absorbance after adding phenylbutazone dropped by 25% (Figure 3D). This indicates that PSNPs do not affect the binding sites of TCS on HSA, but they influence the binding of TCS and HSA by altering the microenvironment and conformation of HSA [73]. Additionally, previous findings suggest that characteristic amino acids near site I play a major role in the relative esterase activity of HSA [31]. The primary reason for the decrease in HSA esterase activity is that PSNPs disrupt the conformation of HSA, thereby affecting the microenvironment of characteristic amino acids near site I and inhibiting the binding of substrates to these residues.

#### 3.3.3. Synchronous Fluorescence Analysis

Synchronous fluorescence spectroscopy is an effective method for studying protein conformational changes induced by small molecules, as it provides information about the microenvironment around amino acid residues [74,75]. Synchronous fluorescence spectroscopy is scanned by fixing the difference between the excitation and emission wavelengths (Δλ = λ_ex_ − λ_em_) [66]. Changes in the microenvironments of Tyr and Trp residues can be observed, when Δλ is set to 15 nm or 60 nm, respectively [52,76].

**Figure 4:**
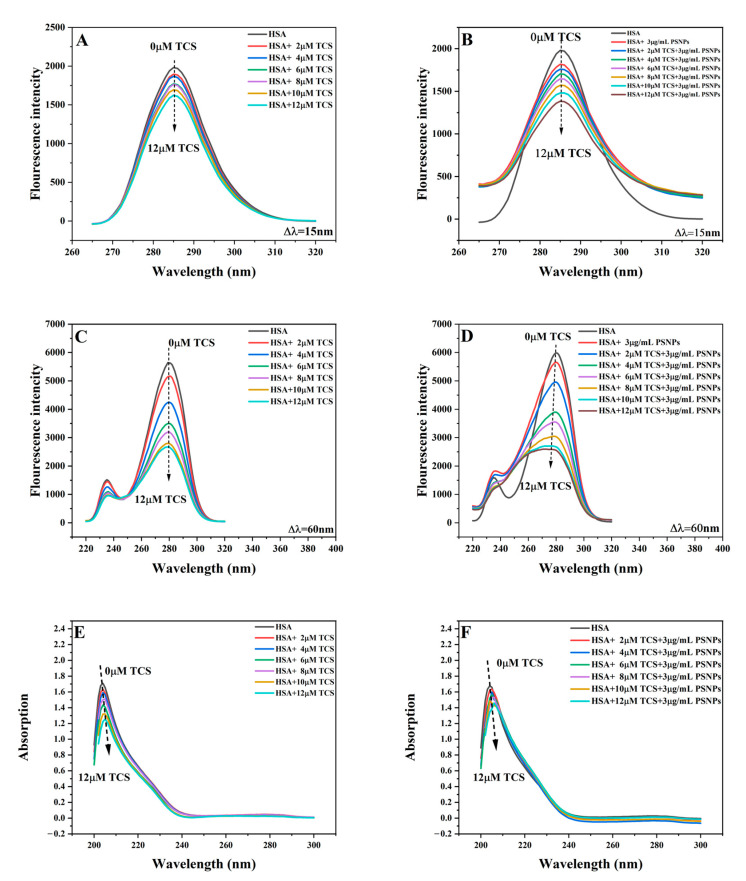
Synchronous fluorescence (**A**–**D**) and UV-vis (**E**,**F**) spectra of HSA-TCS interactions in the absence and presence of PSNPs. [HSA] = 1 μM and T = 293 K.

In the HSA-TCS system, the fluorescence intensities of Tyr and Trp decreased with increasing TCS concentration (Figure 4A,C). However, their maximum emission wavelengths remained unchanged at 285 and 280 nm, indicating that TCS quenched the fluorescence of aromatic amino acids without altering the microenvironment of the amino acid residues. PSNPs exhibited a significant quenching effect on HSA (Figure 4B,D), consistent with previous research [45]. In the HSA-TCS-PSNPs system, both Tyr and Trp showed regular quenching with increasing TCS concentration (Figure 4B,D), and the presence of PSNPs facilitated the quenching of amino acid residues. The maximum emission wavelength of Tyr remained unchanged at 285 nm, indicating that PSNPs had little effect on the microenvironment near Tyr (Figure 4B). In contrast, the maximum emission wavelength of Trp exhibited an 11 nm blue shift (Figure 4D), suggesting that PSNPs changed the microenvironment of Trp, increasing its polarity and reducing its hydrophobicity [13,77]. Changes in the amino acid microenvironment of HSA induced by PSNPs further enhance the binding of TCS and HSA, thereby inhibiting the HSA esterase activity.

#### 3.3.4. UV–Vis Absorption Spectra Investigations

UV-vis absorption spectroscopy is crucial for studying the interaction between small molecules and proteins [78]. HSA exhibits two distinct peaks at 208 nm and 280 nm, corresponding to the backbone peptide chain and amino acid residues, respectively (e.g., Tyr, Trp, and Phe) [23,56].

In the HSA-TCS system, the strong absorption peak near 208 nm was quenched and exhibited a red shift of 1 nm with increasing TCS concentration, while the faint absorption peak near 280 nm remained unchanged (Figure 4E). These suggest that TCS disrupted the hydrogen bonds within the HSA polypeptide chain, leading to the local unfolding of the polypeptide chain and alterations in the protein’s backbone structure without markedly impacting the microenvironment surrounding the amino acid residues [31,79]. In the HSA-TCS-PSNPs system, the absorption peak at 280 nm showed a 6% increase in quenching degree (Figure 4F), indicating that PSNPs affected the microenvironment of the amino acid residues [45]. The quenching degree of the strong absorption peak at 208 nm increased by 11%, and a redshift of 2 nm was observed (Figure 4F). An increase in redshift suggests that PSNPs further unfolded the backbone of the HSA peptide chain, disrupting the structure of HSA and making the amino acid residues more hydrophobic [13,77].

#### 3.3.5. 3D Fluorescence Analysis

**Figure 5:**
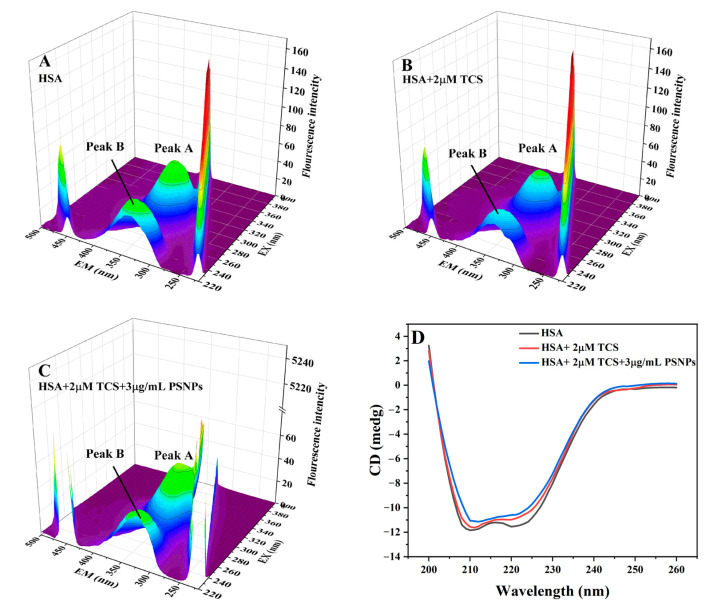
Three-dimensional fluorescence (**A**–**C**) and circular dichroism (**D**) spectra of HSA in the absence and presence of TCS and PSNPs. [HSA] = 1 μM and T = 293 K.

The 3D fluorescence spectroscopy reveals changes in protein structure by observing alterations in the position and intensity of fluorescence peaks [80]. By comparing the 3D spectra of HSA in the absence and presence of TCS and PSNPs, changes in HSA conformation and the microenvironment were examined (Figure 5A–C).

The characteristic peaks for amino acid residues and the protein-peptide chain backbone are represented by the two prominent peaks in the figure, peak “A” (λ_ex_/λ_em_ = 280/331 nm) and peak “B” (λ_ex_/λ_em_ = 226/331 nm), respectively. Peaks “A” and “B” are sensitive to changes in the surrounding environment of amino acid residues. The relevant characteristic values for each peak are listed in Table 2. The fluorescence intensities of the two peaks in the presence of TCS dropped by 15% and 19%, respectively. Peak “B” exhibited a blue shift of 3 nm, and its stokes shift (Δλ) was reduced by 2 nm (Figure 5A,B and Table 2). This indicates that the interaction between TCS and HSA leads to a slight unfolding of protein peptides, which causes TCS to destroy the backbone peptide chain of HSA and change its conformation [43]. In the presence of TCS and PSNPs, the fluorescence intensities of the two peaks decreased by 19% and 25%, respectively (Figure 5A,C and Table 2). Moreover, there was a blue shift of 3 nm in peaks “A” and 6 nm in peaks “B”, with the stokes shift of both peaks reduced by 3 nm (Figure 5A,C and Table 2). This suggests that the microenvironment of the aromatic amino acids was impacted by PSNPs, which decreased the hydrophobicity of the area around them and promoted the stretching of the HSA backbone, leading to a more significant impact on the conformation of HSA [13,52].

#### 3.3.6. CD Spectroscopy Studies

CD spectroscopy is crucial for studying protein conformation and secondary structure changes [81,82]. To investigate the changes in the secondary structure of HSA induced by TCS and PSNPs, the CD spectra of HSA in the absence and presence of TCS and PSNPs were recorded (Figure 5D). The corresponding changes in the secondary structure content were recorded in Table 3.

Two negative peaks at wavelengths of 208 nm and 222 nm are characteristic absorption peaks of α-helices, mainly due to the occurrence of the n → π* leaps in the peptide bonding backbone of HSA [83,84]. In the presence of TCS and the coexistence of TCS and PSNPs, the two negative peaks displayed a growing tendency, while the shape and position of the peaks remained unchanged (Figure 5D). The CD spectra of HSA retained a similar shape in all conditions, indicating that the small molecule bounded to HSA also has an α-helix as their basic structure [85]. The BeStSel method was used to calculate the secondary structure content of HSA [86]. The content of α-helix and β-sheet in HSA was 50.7% and 11.6%, respectively, which reduced to 43.6% for α-helix and 7.9% for β-sheet in the presence of TCS. The decrease in the α-helix and β-sheet content suggests that the interaction of TCS with amino acid residues in the main peptide chain of HSA disrupts hydrogen bonding and locally stretches the HSA peptide chain [77]. In the HSA-TCS-PSNPs system, the α-helix content decreased further to 41.3%, indicating that PSNPs further damaged the hydrogen bonding on the main peptide chain of HSA, exacerbated the unfolding of the peptide chain of HSA and further damaged the protein structure of HSA [13,45]. Prior experimental findings indicate a strong correlation between the secondary structure of proteins and their biological activity [52]. Significant reduction of the α-helix content (from 50.7% to 41.3%) in the presence of TCS and PSNPs suggests that the conformation of HSA was continuously disrupted, thereby affecting HSA esterase activity [63]. The β-sheet content initially decreased and subsequently increased, further elucidating the conformational disruption of HSA induced by TCS and PSNPs. The observed increase in β-sheet content following the addition of PSNPs is hypothesized to be associated with the minor adsorption of HSA by PSNPs. A portion of HSA may become stably bound within PSNPs, leading to an elevated β-sheet content [39,87]. The biological activity of HSA is subject to gradual alterations upon exposure to TCS and PSNPs, highlighting potential environmental impacts [52].

## 4. Conclusions

This study utilized spectroscopic techniques and molecular simulation systems to investigate the interactions between HSA and TCS in the presence of PSNPs. The results show that the esterase activity of HSA was inhibited by increasing concentrations of TCS, and the reduction was more pronounced in the presence of PSNPs. The presence of PSNPs induced the HSA microenvironment of the amino acid residues and conformational changes, which increased the accessibility of the TCS-HSA binding site and enhanced the binding of TCS to HSA. Structural analysis revealed a decrease in the α-helix content of HSA after interacting with TCS and PSNPs, indicating that these interactions disrupted the secondary structure of the protein. This disruption is likely due to the synergistic effect of PSNPs and TCS, thereby exacerbating the unfolding of the HSA polypeptide chain and affecting its biological activity. The findings demonstrate the potential synergistic toxicity of PSNPs and TCS at the molecular level, highlighting the crucial for further research into the toxicological effects of such complex pollutants. Future studies should synthesize multiple biological levels to collectively assess the toxicity of complex pollutants to proteins since various physiological mechanisms affect the biological function of proteins.

## Figures and Tables

**Table 1:** Quenching constants for the HSA-TCS system and HSA-TCS-PSNPs system at different temperatures.

System	T (K)	K*_SV_* (×10^7^ M^−1^)	K*q* (×10^15^ M^−1^ s^−1^)	R^2^
HSA-TCS	273	4.03	4.03	0.991
	293	3.33	3.33	0.990
	313	1.96	1.96	0.991
HSA-TCS-PSNPs	273	4.35	4.35	0.992
	293	3.52	3.52	0.993
	313	2.58	2.58	0.992

**Table 2:** HSA characteristics in the absence and presence of TCS and PSNPs.

System	Peak	Peak Position [λ_ex_/λ_em_ (nm/nm)]	Intensity
HSA	Peak A	280/331	64.53
	Peak B	226/331	58.48
HAS + TCS	Peak A	280/331	54.57
	Peak B	226/328	47.49
HAS + TCS + NPs	Peak A	280/328	52.10
	Peak B	226/325	43.50

**Table 3:** Proportions of different secondary structures of HSA in the absence and presence of TCS and PSNPs.

System	α-Helix	β-Sheet	β-Turns	Other
HSA	50.7	11.6	11.6	27.4
HAS + TCS	43.6	7.90	10.4	32.1
HAS + TCS + PSNPs	41.3	12.1	9.40	26.7

## Data Availability

Data will be made available upon request.
